# Preparation and spectroscopic characterization of lyophilized Mo nitrogenase

**DOI:** 10.1007/s00775-020-01838-4

**Published:** 2020-12-30

**Authors:** Casey Van Stappen, Laure Decamps, Serena DeBeer

**Affiliations:** Max Planck Institute for Chemical Energy Conversion, Stiftstrasse 34-36, 45470 Mülheim an der Ruhr, Germany

**Keywords:** Nitrogenase, X-ray absorption spectroscopy, Electron paramagnetic resonance, Lyophilization

## Abstract

**Abstract:**

Mo nitrogenase is the primary source of biologically fixed nitrogen, making this system highly interesting for developing new, energy efficient ways of ammonia production. Although heavily investigated, studies of the active site of this enzyme have generally been limited to spectroscopic methods that are compatible with the presence of water and relatively low protein concentrations. One method of overcoming this limitation is through lyophilization, which allows for measurements to be performed on solvent free, high concentration samples. This method also has the potential for allowing efficient protein storage and solvent exchange. To investigate the viability of this preparatory method with Mo nitrogenase, we employ a combination of electron paramagnetic resonance, Mo and Fe K-edge X-ray absorption spectroscopy, and acetylene reduction assays. Our results show that while some small distortions in the metallocofactors occur, oxidation and spin states are maintained through the lyophilization process and that reconstitution of either lyophilized protein component into buffer restores acetylene reducing activity.

**Graphic abstract:**

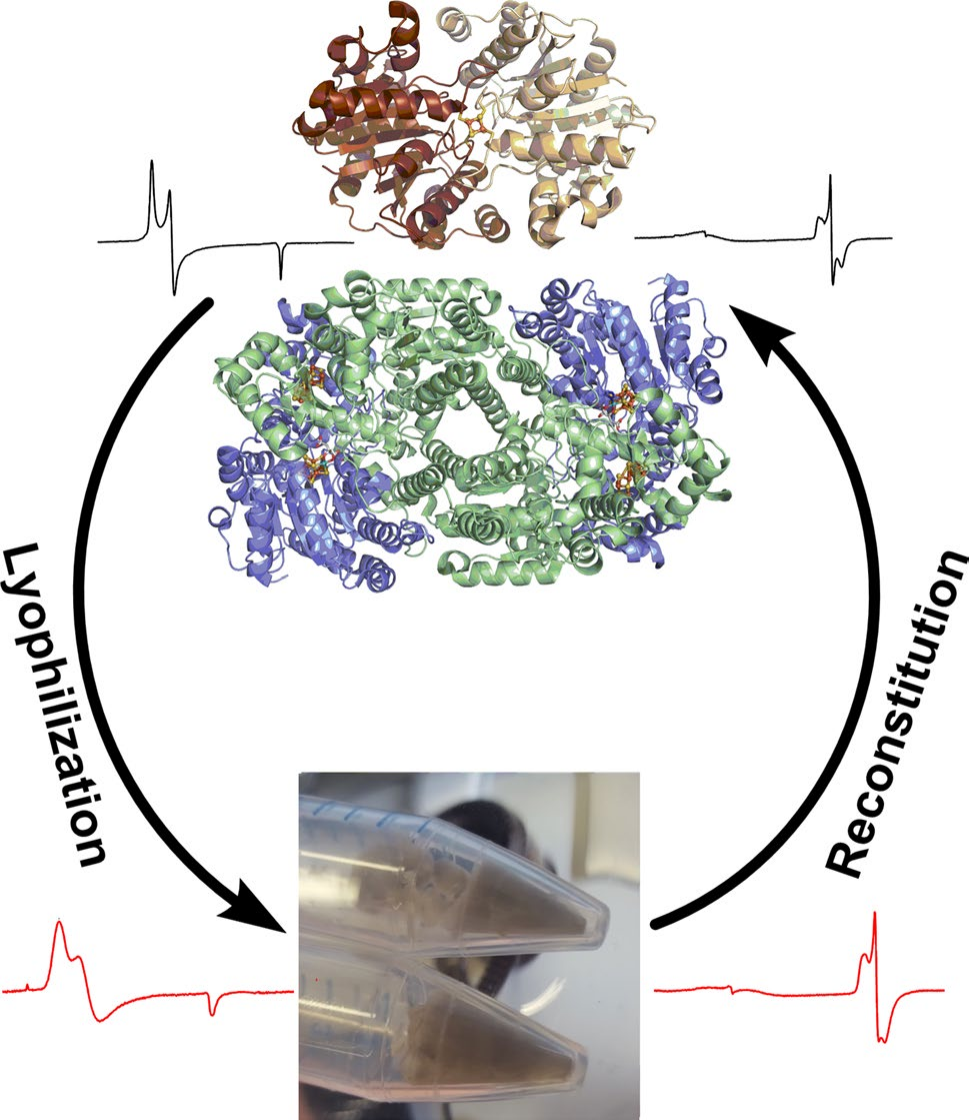

**Supplementary Information:**

The online version contains supplementary material available at 10.1007/s00775-020-01838-4.

## Introduction

**Fig. 1:**
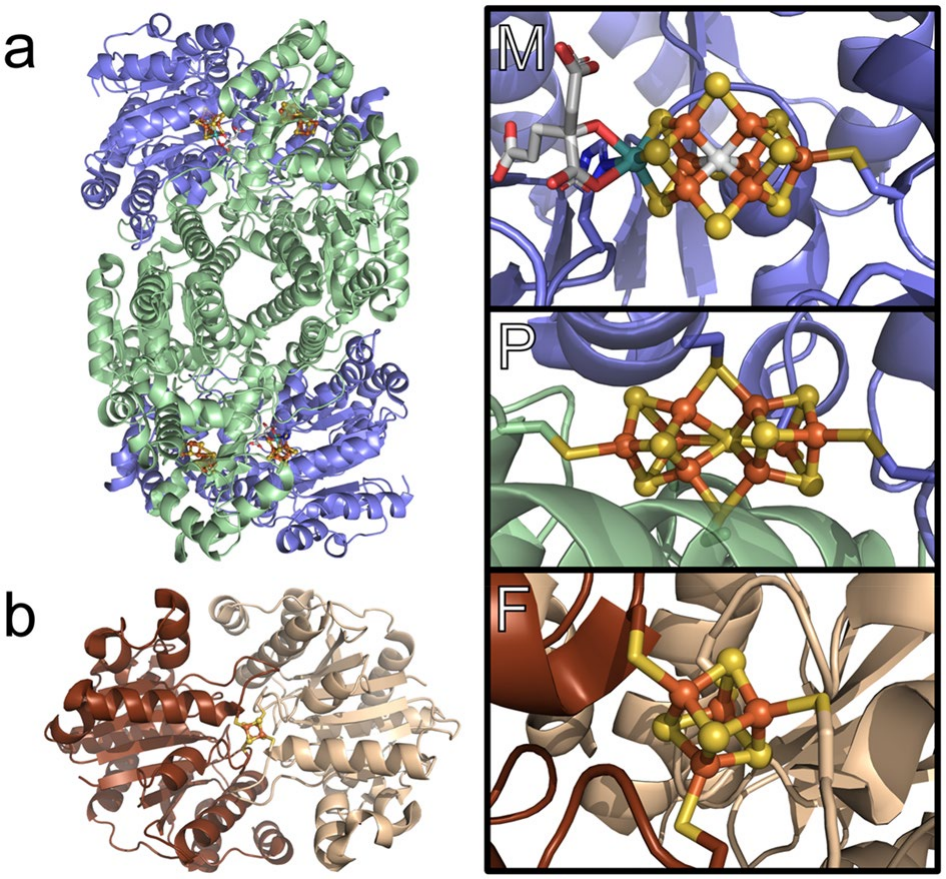
The **a** MoFe and **b** FeP proteins of Mo nitrogenase, and their corresponding metallocofactors, the M-cluster (FeMoco), P-cluster, and F-cluster. The *α*-subunit of MoFe is displayed in blue, while the *β*-subunits are displayed in green (PDB 3U7Q) [20]. The two symmetric *α*-subunits of FeP are displayed in tan and brown (PDB 6N4K) [21]

Conversion of N_2_ to NH_3_ is a critical biogeochemical process and essential for life on earth. In biology, this reaction is carried out exclusively by a small family of nitrogenase enzymes. Nitrogenases are two component systems, consisting of a catalytic *M*Fe protein (*M* = Mo, V, Fe) and a corresponding iron–sulfur cluster containing protein (FeP), which serves as the physiological reductant of *M*Fe [1–8]. Of the three known nitrogenases, the Mo-dependent enzyme is by far the widest spread, with production of the V and Fe homologs only appearing in certain organisms under Mo-limiting conditions [9, 10]. As a result, the study of these enzymes has heavily focused on the Mo-dependent nitrogenase [11–19]. To accomplish this reaction, Mo nitrogenase employs a series of iron–sulfur metallocofactors are employed, specifically the catalytic M-cluster (FeMoco) and P-cluster of MoFe, and the [4Fe–4S] F-cluster of FeP, shown in Fig. 1.

Although extensive spectroscopic, crystallographic, and reactivity studies have helped to shed light on the elusive mechanism of Mo nitrogenase, much still remains to be understood [15–19, 22]. A significant limitation in studying this enzymatic system is the concentrations that can be reached before these proteins precipitate in solution. This has generally limited the applicability of spectroscopic techniques requiring extremely high metal concentrations, such as soft X-ray absorption and emission methods. In addition, the processes required for achieving high protein concentrations in solution, such as centrifugal filtration, can also result in significant protein losses from denaturation or aggregation [23, 24]. Given that production of nitrogenases for study still requires expression through native organisms such as *Azotobacter vinelandii* or *Clostridium pasteurianum*, methods for preparing Mo nitrogenase in the high concentrations required for many spectroscopic techniques with minimal protein loss are highly desirable.

Lyophilization is a common preparatory technique for storing proteins and even whole cells. This process involves deep freezing of a solution of protein or cells to form a frozen matrix, followed by application of a vacuum to the headspace to remove water through sublimation [25, 26]. In this way, all unbound water can be removed while hypothetically minimally perturbing the protein itself and avoiding agglomeration. The resulting protein substrate then represents the maximal achievable concentration of the protein, consisting of only the protein matrix, bound water, and whatever salts may have been included in solution prior to lyophilization. Concentrations can be pushed even further by desalting proteins and using labile buffers, such as ammonium formate or ammonium acetate, which are also removed by vacuum during the lyophilization process. This preparatory technique has been used particularly heavily in solid-state NMR studies to investigate protein structure, sedimentation and solvation effects, and even metabolic pathways in whole cells [27–30].

Although lyophilization provides a promising route to obtaining a theoretical maximum protein concentration without loss of protein mass, the viability of the lyophilized substrate for direct study or reconstitution is not always guaranteed. The process itself can result in protein degradation through oxidation, deamidation, and aggregation [25, 26]. These issues can arise from a range of factors in the lyophilization process, including fluctuations in pH changes during freezing, ice-exclusion effects resulting in rapid local concentration increases, cold denaturation, and dehydration stress [25, 26, 31, 32]. Before using lyophilization as a sample preparation method for either advanced spectroscopy or protein storage, it is important to establish the viability of this method for the specific protein or system of interest by characterizing the properties of the lyophilized and reconstituted states.

Previously, lyophilized preparations of MoFe have been investigated in both hard and soft X-ray applications [33–36]. Mo K-edge XAS has been reported comparing the lyophilized form of MoFe extracted from *Clostridium pasteurianum* with that of crystallized MoFe from *Azotobacter vinelandii*. These early studies suggested that lyophilization results in some small perturbations of the coordination environment of Mo, but that the overall oxidation state is maintained [35]. Fe 2*p*3*d* XMCD has also been reported, demonstrating the suitability of this preparatory method for studying MoFe under ultra-high vacuum conditions [33]. Associated EPR measurements showed that the ground *S* = ^3^/_2_ spin state, attributed to the resting FeMoco cluster, is maintained through the process of lyophilization and reconstitution [33].

Although previous studies have utilized lyophilization as a preparatory method for studying both whole bacterial cells of nitrogen-fixing organisms as well as extracted MoFe, it is still unclear how this technique impacts the metallocofactors of MoFe and FeP electronically and structurally, particularly when prepared under salt-free conditions using labile buffers. Herein, we report a more complete spectroscopic characterization of the metallocofactors of lyophilized Mo nitrogenase using a combination of EPR and both Mo and Fe K-edge X-ray absorption spectroscopies. Furthermore, we report the catalytic properties of the reconstituted forms of the two isolated protein components, MoFe and FeP, demonstrating the catalytic capacities of both proteins are retained through the lyophilization/reconstitution process.

## Materials and methods

### Protein preparation, lyophilization, and reconstitution

Nitrogenase proteins MoFe and FeP were prepared as previously described [37]. Protein concentrations were determined by Lowry assay [38]. Protein purities were determined to be > 95% based on SDS-PAGE analysis with Coomassie staining. To prepare for lyophilization, solutions of MoFe and FeP in 50 mM Tris, 200 mM NaCl at pH 7.4 were desalted and exchanged into a 50 mM ammonium formate buffer at pH 7.4 using a Sephadex™ G-25 gel filtration column. The resulting solutions were frozen in liquid N_2_, placed in Schlenk glassware, and lyophilized at an average pressure of 1 × 10^–4^ bar for 48 h using a Martin Christ Alpha 1–4 LOC-1 M lyophilizer. Reconstitution of the resulting lyophilizates for EPR measurements and activity assays was performed by addition of a buffer solution containing 20 mM Tris (pH  7.3), 100 mM NaCl, and 2.5 mM sodium dithionite followed by gentle shaking. All manipulation of proteins and buffers were performed under a 98% N_2_, 2% H_2_ atmosphere.

### Acetylene reduction assay

Nitrogenase activity assays for C_2_H_2_ reduction were performed for solutions of MoFe and FeP both prior to and following lyophilization. Assays were prepared under an Ar atmosphere as 1 ml solutions in sealed 10 ml vials containing 5 μM MoFe, 10 μM FeP, 0.4 mg/ml creatine phosphokinase, 60 mM creatine phosphate, and 10 mM sodium dithionite dissolved in a buffer solution of 20 mM Tris buffer, 100 mM NaCl, and 10 mM MgCl_2_ calibrated to pH 7.4. A 1 ml volume of C_2_H_2_ at 1 atm was transferred to each sealed vial, and equilibrated at 30 °C for 5 min with gentle shaking. The assays were initiated by the addition of 20 μl of MgATP (2.5 mM final concentration), followed by incubation at 30 °C for 30 min with gentle shaking. All reactions were terminated by the addition of 250 μl glacial acetic acid. The production of C_2_H_4_ was quantified by gas chromatography using a flame ionization detector fitted to a Shimadzu GC-2010 Plus ATF gas chromatograph with a 30 m long RT®-Q-Bond column with 20 μm internal diameter, using He as a carrier gas. Gas chromatograms were collected and analyzed using LabSolutions software (version 5.92). No C_2_H_6_ was observed.

### Electron paramagnetic resonance

EPR spectra were recorded using a Bruker X-band ESP 300 spectrometer equipped with a Bruker dual-mode cavity (ER4116DM) and an Oxford Instruments ESR 900 continuous flow cryostat. Measurements utilized a 2 mW microwave power and 0.746 mT/100 kHz modulation at temperatures of 10 K. Spectra were analyzed using the software package EasySpin (version 5.1.9) as implemented in Matlab [39]. MoFe^rec^ and FeP^rec^ were prepared by reconstitution of the lyophilized proteins into a buffer solution containing 20 mM Tris, 100 mM NaCl, and 2.5 mM dithionite (pH 7.3). MoFe^lyo^ and FeP^lyo^ were transferred into X-band EPR tubes manually using a custom-fitted funnel. All EPR sample preparations were performed under an anoxic N_2_ atmosphere.

### X-ray measurements

X-ray absorption measurements of intact nitrogenase MoFe in solution were obtained at the 9–3 beamline of the Stanford Synchrotron Radiation Lightsource (SSRL). The SPEAR storage ring operated at 3.0 GeV in top-off mode with a 500 mA ring current. A liquid N_2_ cooled double-crystal monochromator using Si(220) crystals at *ϕ* = 0° was used to select the incoming X-ray energy with an intrinsic resolution (Δ*E/E*) of 0.6 × 10^–4^, and a Rh-coated mirror was used for harmonic rejection. The X-ray beam size was 1 × 4 mm^2^ (*V* × *H*) at the sample position. A liquid helium flow cryostat was used to maintain the sample environment at approximately 20 K to prevent radiation damage and maintain an inert sample environment. Fluorescence measurements were recorded using a Canberra 100-element Ge monolith solid-state detector. Before measurements, each sample was checked for signs of radiation damage by performing subsequent 5-min scans over the same sample spot. These tests showed the MoFe was stable under X-ray irradiation at the Mo K-edge for > 90 min, and > 70 min at the Fe K-edge.

X-ray absorption measurements of lyophilized MoFe protein were obtained at the P64 beamline of the Positron–Electron Tandem Ring Accelerator (PETRA III) at the Deutsches Elektronen-Synchrotron (DESY). Samples were prepared as solids, held between two layers of Kapton tape in a 1 mm thick aluminum spacer. The storage ring operated at 6.0 GeV in top-off mode with a ~ 100 mA ring current. A liquid nitrogen cooled double-crystal monochromator using pairs of either Si(311) or Si(111) crystals (at the Mo and Fe K-edges, respectively) were used to select the incoming X-ray energy with intrinsic resolutions (Δ*E/E*) of ~ 1.4 × 10^–4^ and 0.3 × 10^–4^, and a Rh-coated mirror was used for harmonic rejection. The X-ray beam size was 1.5 × 0.5 mm^2^ (*V* × *H*) at the sample position. A liquid helium closed-cycle cryostat was used to maintain a ~ 15 K sample environment in order to minimize radiation damage and maintain an inert sample environment. Fluorescence measurements were recorded using a Vortex®-EM silicon drift detector with an additional a Z-2/Z-1 high-pass filter at Mo and Fe K-edges, respectively. Prior to measurement, each sample was checked for signs of radiation damage by performing subsequent 5-min scans over the same sample spot. These tests showed the MoFe protein was stable under X-ray irradiation at the Mo K-edge for > 60 min, and > 30 min at the Fe K-edge, similar to that of MoFe in solution.

For Mo XAS measurements, the energy of the incoming X-rays was calibrated by simultaneous measurement of a Mo foil and assigning the energy of the maximum of the white line to 20,016.4 eV. Full XAS scans were collected by scanning the incident energy from 19,780 to 21,142 eV. All Fe XAS measurements were collected by scanning the incident energy from 6882 to 8093 eV and calibrated by simultaneous measurement of an Fe foil, with the first inflection point set to 7111.2 eV.

### X-ray data analysis

In all experiments, individual scans were normalized to the incident photon flux and averaged using the program Athena from the software package Demeter [40]. Further processing of spectra including background subtraction and normalization was also performed using Athena, following standard protocols for X-ray spectroscopy described below. EXAFS fitting was performed using the program Artemis, also of the software package Demeter [40]. Possible scattering paths for the EXAFS models were initially determined using FEFF 6.0 in combination with a recent high-resolution crystal structure (PDB ID 3U7Q) [20, 41]. The structural parameters *R* (bond distance) and *σ*^*2*^ (bond variance) were allowed to vary during fitting refinement for all measured data. A value of S_0_^2^ of 1 was used in fitting the Mo EXAFS, and 0.9 in fitting the Fe EXAFS. A single Δ*E*_0_ parameter was assigned to all scattering paths at a given edge, and allowed to vary in the refinement of the resting state Mo and Fe K-edge EXAFS.

### Mo data processing and EXAFS fitting

Background subtraction and normalization of the averaged Mo EXAFS spectrum was performed using a linear regression for the pre-edge region of 19,910–19,947 eV, and a quadratic polynomial regression for the post-edge region of 20,157–20,807 eV. The data were splined from *k* = 0–17.2 Å^−1^ using an R-background of 1.0 Å and k-weight of 2. Both *k*^2^ and *k*^*3*^ weighting were used to balance the importance of data at high and low *k*. A *k*-range of 2–14 Å^−1^ was used for the curve fitting analysis of both MoFe^pre^ and MoFe^lyo^ giving a maximum resolution of Δ*R* = 0.13 Å. All data were fit in *R*-space using an *R*-range of 1.5 to 3.5 Å. Owing to the already considerable complexity of the EXAFS of the MoFe protein, fitting was limited to include only single scattering paths, and the degeneracies of these paths, *N*_i_, were fixed to previously established values based on crystallography [20]. No smoothing was used at any point in any of the data processing.

Approaches to fitting the Mo K-edge EXAFS of MoFe have been previously described in detail [42], and presently we have employed a model involving Mo–Fe, Mo–S, and Mo–O single scattering paths based on the high-resolution crystal structure of resting MoFe [20]. Although it has been established that resting MoFe is coordinated by one N from *α*His442 and two O from homocitrate [20], the similar scattering properties of O and N make them undifferentiable with the fidelity of the present spectral data. Therefore, the employed Mo–O scattering pathway in our model represents an amalgamation of both Mo–O and Mo–N coordination, and will referred to Mo–O/N.

### Fe data processing and EXAFS fitting

The Fe EXAFS was processed in a similar fashion to that of the Mo EXAFS. Background subtraction and normalization was performed using a linear regression for the pre-edge region of 6990–7005 eV, and a quadratic polynomial regression for the post-edge region of 7160–8200 eV. The data were splined from *k* = 0–15.9 Å^−1^ using an *R* background of 1.0 and *k* weight of 2. A *k*-range of 2–12 Å^−1^ was used in the curve fitting analysis of samples of MoFe both before and following lyophilization, providing a maximum resolution of Δ*R* = 0.16 Å. All data were fit in *R*-space using an *R*-range of 1.5 to 4.0 Å. Owing to the already considerable complexity of the EXAFS of the MoFe protein, fitting was limited to include only single scattering paths involving Fe–Mo, Fe–S, split short Fe–Fe, and long Fe–Fe scatterers. Accompanying degeneracies, *N*_i_, for each of these five pathways were fixed based on previous established values from EXAFS and XRD studies [20, 42, 43]. Although the presence of a hypervalent C at the center of FeMoco has been established through other spectroscopic means [20, 44], we have previously demonstrated that the Fe–C scattering pathway does not provide any statistical improvement to the model [42]. Therefore, this scattering path has been excluded from the present fitting models in an effort to minimize the number of free parameters. In order to help further minimize the number of free parameters, the bond variance parameters (*σ*^2^) for each of the two short Fe–Fe scattering paths were fixed to be equivalent and simultaneously fit. This is a reasonable approximation as the scatterers are of the same identity and in a similar environment. No smoothing was used at any point in any of the data processing.

## Results and discussion

The MoFe and FeP proteins were investigated in three states: (a) prior to lyophilization, (b) lyophilized, (c) and following reconstitution of lyophilized protein into buffer solution. The superscript notations “pre”, “lyo”, and “rec” adjacent to the protein name are used to distinguish between these three respective states. For example, the lyophilized state of MoFe is referred to as MoFe^lyo^, while reconstituted lyophilized MoFe is referred to as MoFe^rec^.

**Fig. 2:**
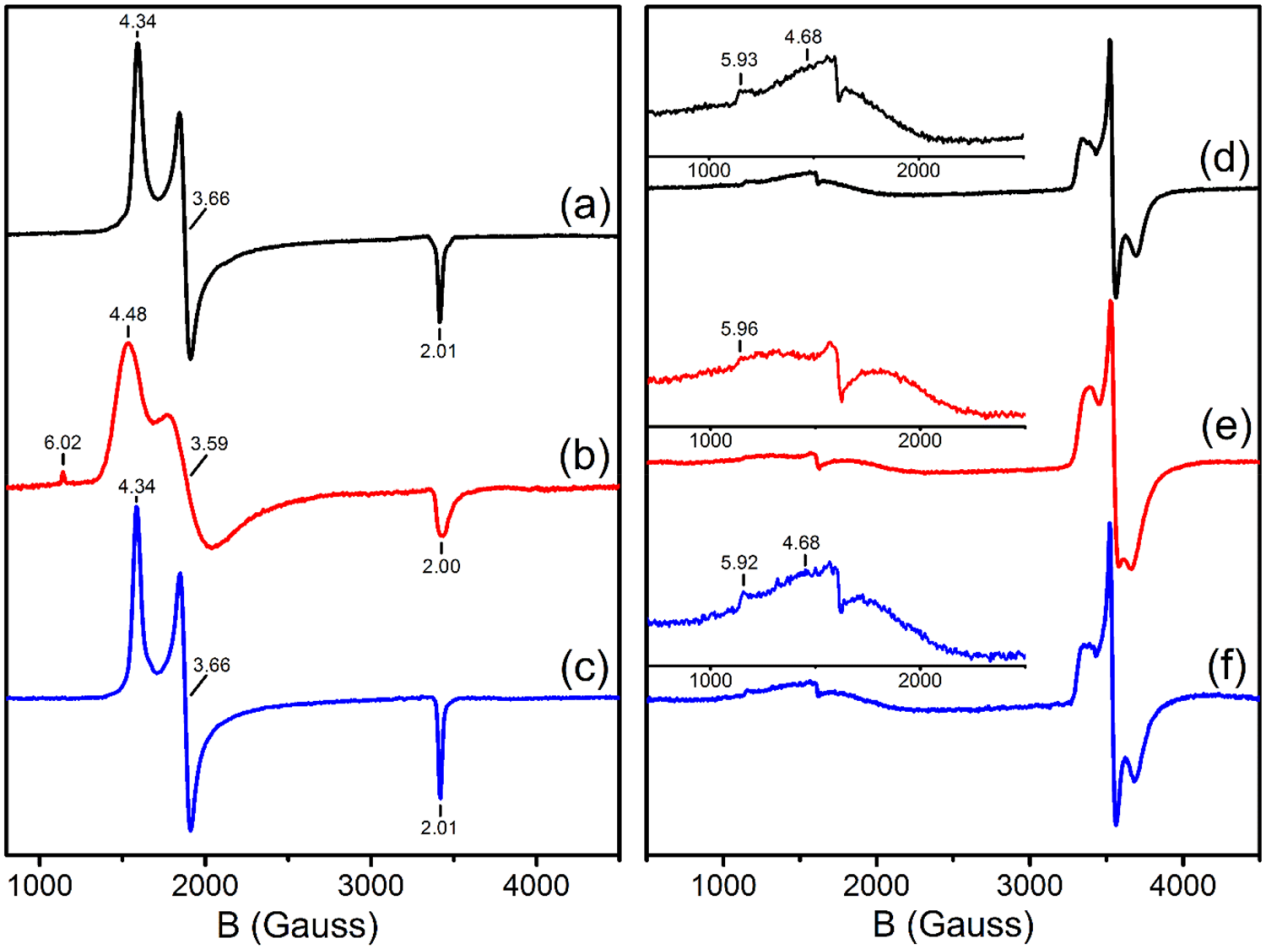
X-band CW perpendicular-mode EPR spectra of (left) MoFe and (right) FeP. **a** MoFe^pre^, **b** MoFe^lyo^, **c** MoFe^rec^, **d** FeP^pre^, **e** FeP^lyo^, and **f** FeP^rec^. Insets of the low field region for samples of FeP are provided to highlight the *S* = ^3^/_2_ signals. Measurements were performed at 10 K, 9.6 GHz using a power of 2 mW and a 7.46 G modulation amplitude

To probe the magnetic properties of MoFe and FeP through the lyophilization and reconstitution process, X-band EPR measurements were performed. The perpendicular mode spectra are summarized in Fig. 2, and Table 1 contains a summary of fit spectral parameters. Meanwhile, no signals were observed in the parallel mode EPR for either MoFe or FeP in any of the three discussed states. In the perpendicular mode EPR, MoFe^pre^ produces the well-characterized inflections at *g*_eff_ = [4.34, 3.66, 2.01] of the E_0_ resting state of FeMoco, which correspond to the magnetic transitions within the *m*_S_ =  ± ^1^/_2_ manifold of the *S* = ^3^/_2_ system [45]. MoFe^lyo^ produces a similar but with increased rhombicity, extending to *g*_eff_ = [4.48, 3.59, 2.00]. An additional inflection at *g* = 6.02 is also seen, which corresponds to the low-field excited state magnetic transition of the m_S_ =  ± ^3^/_2_ manifold. Based on the determined *g*_eff_ of MoFe^pre^ and MoFe^lyo^, the process of lyophilization increases the rhombicity of the magnetic environment of FeMoco, shifting from *E/D* = 0.056 to 0.074, respectively. In addition, the spectrum of MoFe^lyo^ exhibits a significantly greater degree of broadening. Such broadenings can arise from inhomogeneity in the microscopic environment around the spin center, or from dipole–dipole interactions between neighboring spin centers. All of these changes (increased rhombicity, spectral broadening, and population of the *m*_s_ = ^3^/_2_ manifold) are strikingly similar to previous observations for extracted FeMoco in the presence of 10 mM thiophenol/1 mM sodium dithionite, as well as MoFe in 80% *N*-methylformamide (NMF)/20% 100 mM Tris/Cl at pH 8.0 [46]. However, the latter also presents an additional *S* = ^1^/_2_ signal, which is not present in MoFe^lyo^. As mentioned earlier, no features are observed in the parallel mode EPR, indicating that P^2+^ (which displays a signature inflection at *g* = 11.6–11.8) is not present [19, 47]. Reconstitution of MoFe^lyo^ into 20 mM Tris buffer (pH 7.3) to form MoFe^rec^ produces an *S* = ^3^/_2_ signal near identical to MoFe^pre^, indicating an unperturbed FeMoco cluster (Fig. 2c).

**Table 1:** X-band CW EPR fitting parameters for MoFe and FeP. Plots of corresponding fits are provided in the SI

Sample	*S*	*g*_eff_	*E/D*
MoFe^pre^ MoFe^lyo^ MoFe^rec^	3/2 3/2 3/2	[4.34, 3.66, 2.01] [4.48, 3.59, 2.00] [4.34, 3.66, 2.01]	0.056 0.074 0.056

		*g*	
FeP^pre^ FeP^lyo^ FeP^rec^	1/2 1/2 1/2	[2.05,1.94,1.86] [2.04,1.94,1.87] [2.05,1.94,1.86]	– – –

The behavior of FeP moving between the solution and lyophilized states (Fig. 2d–f) shows a similar pattern. The spectrum of FeP^pre^ displays a well-established spin-admixture of *S* = ^3^/_2_ and ^1^/_2_ signals, which has been previously shown to vary in relative intensity depending on sample environment [48]. Although the *S* = ^3^/_2_ signal beginning ~ *g* = 5.92 is still present in FeP^lyo^, its integrated intensity appears to decrease by ~ 10% when compared with the spectra of either FeP^pre^ or FeP^rec^, with a concomitant increase of ~ 10% in relative intensity of the *S* = ^1^/_2_ signal. This implies there is a small preference for formation of the *S* = ^1^/_2_ ground state upon lyophilization. It is worthwhile to note this shift in spin–admixture composition is considerably less extreme than what has been seen in solution for the addition of possible exogeneous ligands like ethylene glycol or urea [48–50]. The *S* = ^1^/_2_ signal of FeP^lyo^ also appears modestly broadened relative to either FeP^pre^ or FeP^rec^, but somewhat less so than for our observations of MoFe^lyo^. Modulation of the *g* tensors also appears minimal, with best fit values even indicating a slight *decrease* in rhombicity. Therefore, despite the exposed nature of the F-cluster of FeP, lyophilization appears to have a relatively minor impact on the electronic structure of this metallocofactor.

**Fig. 3:**
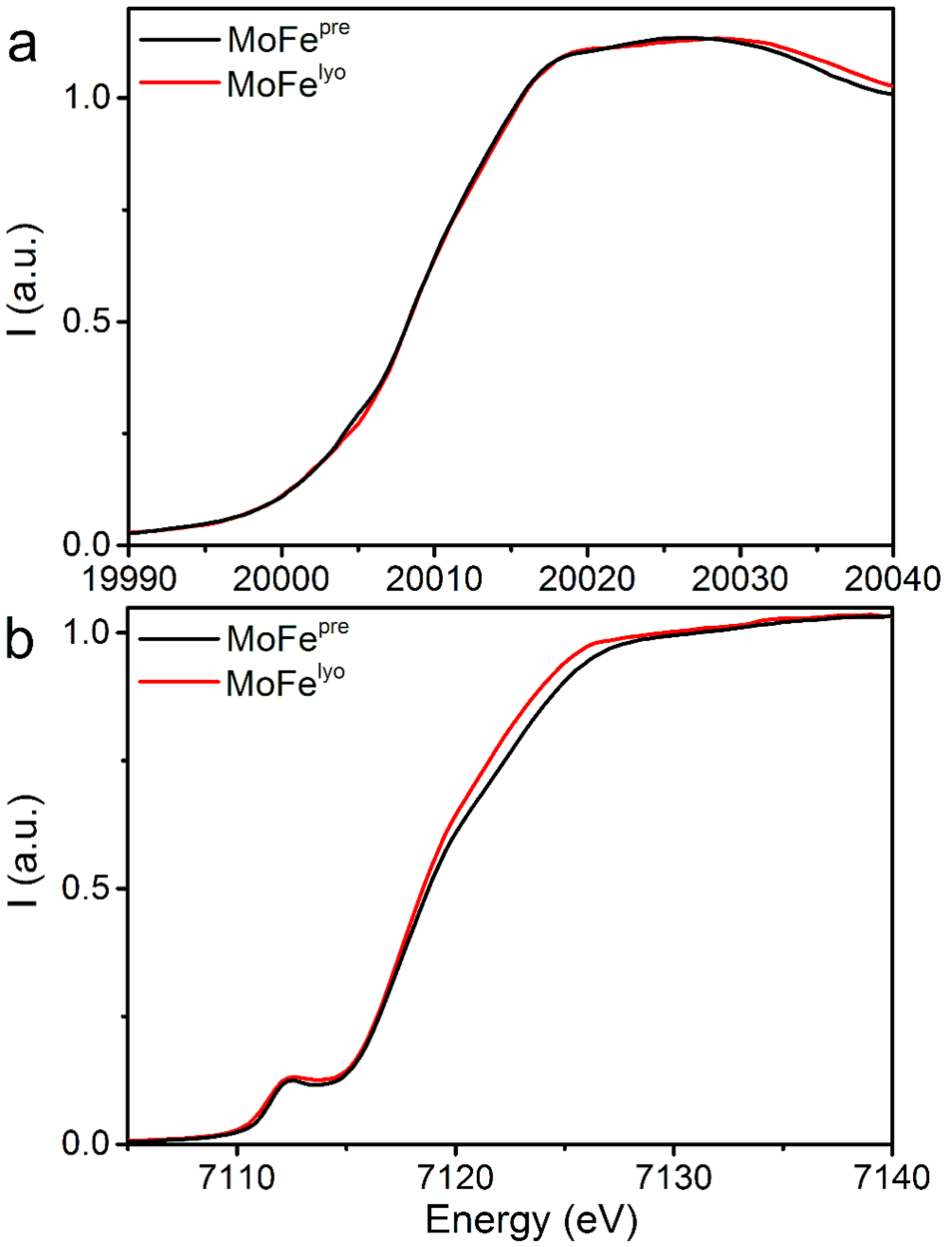
**a** Mo K-edge and **b** Fe K-edge X-ray absorption spectra of MoFe^pre^ (black) and MoFe^lyo^ (red)

Building on our results from EPR, further X-ray absorption measurements were performed to investigate the electronic and structural changes that occur at the metallocofactors of MoFe upon lyophilization. The Mo and Fe K-edge X-ray absorption spectra of MoFe^pre^ and MoFe^lyo^ are compared in Fig. 3. At Mo, no significant changes are observed in terms of either edge position or shape, implying that Mo remains as Mo^III^ in the lyophilized state. This is consistent with previous observations comparing lyophilized and solution-phase MoFe extracted from *Clostridium pasteurianum* [36]. Meanwhile, several changes are observed in the Fe K-edge spectrum. First, a broadening of the pre-edge ~ 7112 eV combined with a small increase in intensity is observed in MoFe^lyo^. Although the rising-edge position appears near-identical from 7115–7117 eV, the edge of MoFe^lyo^ above 7118 eV appears shifted to lower energies. The broadening of the pre-edge is likely a result of the difference in resolution provided by the monochromator crystals used to obtain each set of data (with Si(220) in the case of MoFe^pre^ and Si(111) for MoFe^lyo^). Our previous studies have evidenced that very little spectral change is observed with single e^−^ reduction/oxidation of FeMoco due to the large number of unique Fe in MoFe [42, 51]. Nevertheless, the lack of observable P^1+^ or P^2+^ via EPR combined with the present Fe K-edge XAS results support that MoFe is not oxidized during lyophilization. Computational and experimental studies have implicated that both the structure and degree of electronic delocalization in the P and FeMoco clusters are linked to the surrounding hydrogen bonding environment of the metallocofactors [42, 52–55], and interruption of this network may be responsible for the observed increase in intensity of the upper portion of the edge and white line (7118–7123 eV).

**Fig. 4:**
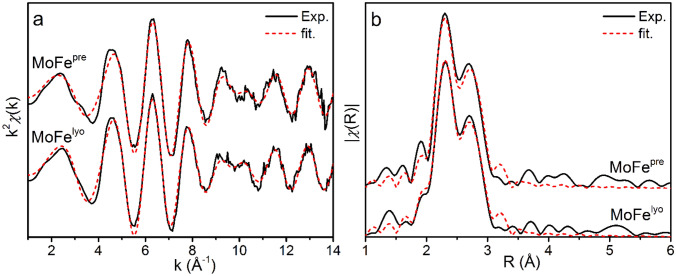
Mo K-edge EXAFS of MoFe^pre^ and MoFe^lyo^ presented in **a** *k*-space and **b** *R*-space, phase shifted to the Mo–S scattering path. Experimental spectra are provided as solid black lines, and corresponding fits as red dashed lines. Spectra are *k*^2^-weighted, and the Fourier transform was performed for a k-range of 0–14 Å. Fits were performed using *k*^2,3^-weighting over ranges of *k* = 2–14 Å^−1^ and *R* = 1.5–3.5 Å

Structural characterization of MoFe^pre^ and MoFe^lyo^ was carried out by Mo and Fe K-edge extended X-ray absorption fine structure (EXAFS) analysis. The Mo K-edge EXAFS spectra of resting MoFe^pre^ and MoFe^lyo^ are provided in Fig. 4, and fitting parameters are summarized in Table 2. Only small modulations in the *k*-space EXAFS spectrum are observed between, predominately between 9–11 Å^−1^. The phase-shifted *R*-space spectra are dominated by two main features at 2.3 and 2.7 Å which are generally attributed to the Mo–S and Mo–Fe scattering paths, respectively. While the feature at 2.3 Å appears unperturbed, a small decrease in absolute intensity is observed at 2.7 Å. This is indicative of a greater degree of disorder in the Mo–Fe scatterer, as seen by a small increase in *σ*^2^ for this scattering path (Table 2). The largest modulation, however, appears to be in a decrease in distance of the light atom Mo–O/N scattering path, which contracts from 2.20 to 2.16 Å upon lyophilization of MoFe. Computational studies have suggested that this distance is extremely sensitive to the hydrogen bonding environment of the homocitrate which binds the Mo of FeMoco and has been proposed to be an integral part of the proton relay pathway during turnover [42, 52–54, 56–58]. Combined with our observations at the Fe K-edge in Fig. 3, this contraction provides further evidence that the hydrogen bonding network surrounding FeMoco is disrupted in the lyophilized state.

**Table 2:** Mo K-edge EXAFS fitting parameters for MoFe^pre^ and MoFe^lyo^. Statistical errors are provided for *σ*^2^ and *R* as determined from the fitting procedure

	Path	*N*^a^	*σ*^2^ (10^–3^ Å^2^)	*R* (Å)	*E*_0_ (eV)	*R*-factor
MoFe^pre^	O/N	3	5.3 (2.2)	2.20 (0.02)	20,011 (1)	0.0170
	S	3	1.5 (0.1)	2.35 (0.01)		
	Fe	3	2.2 (0.4)	2.69 (0.01)		
MoFe^lyo^	O/N	3	8.9 (4.0)	2.16 (0.03)	20,010 (2)	0.0096
	S	3	1.5 (0.5)	2.35 (0.01)		
	Fe	3	2.6 (0.4)	2.70 (0.01)		

For both data sets, fits were performed using *k*^2,3^-weighting over ranges of *k* = 2–14 Å^−1^ and *R* = 1.5–3.5 Å

**Fig. 5:**
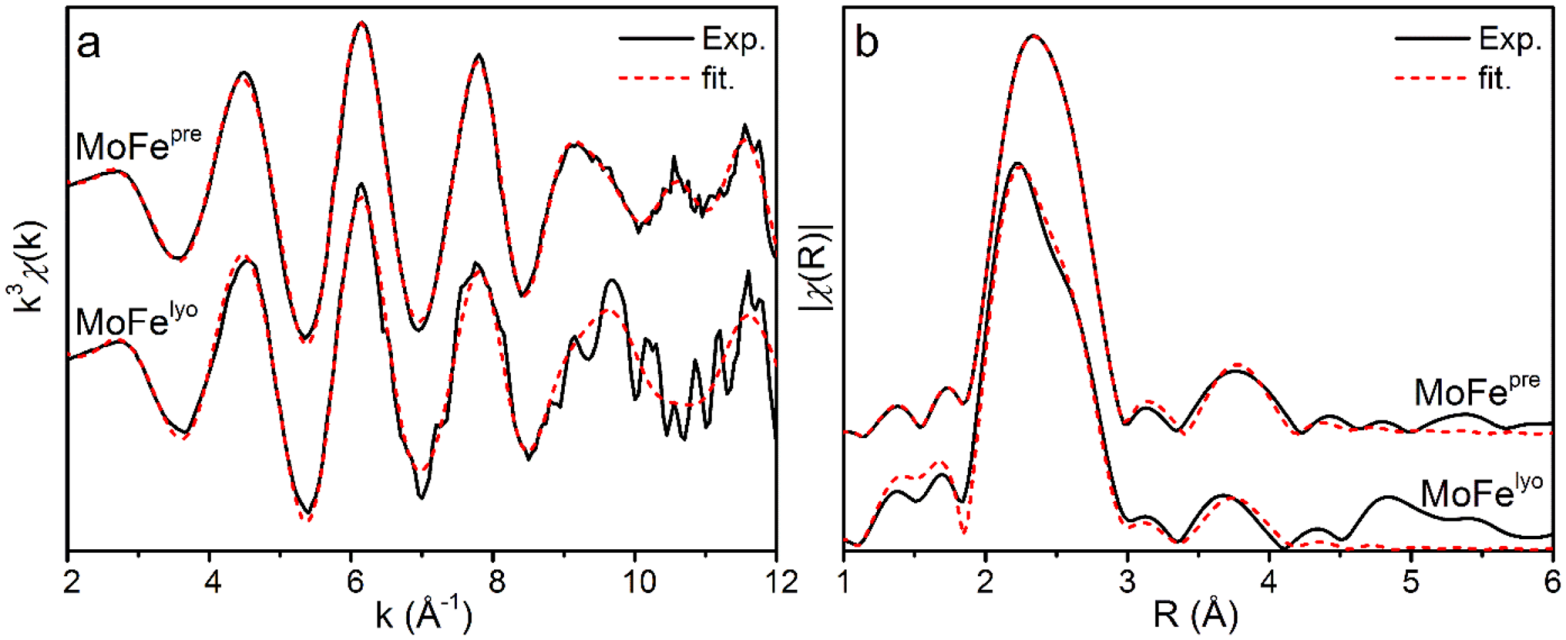
Fe K-edge EXAFS of MoFe^pre^ and MoFe^lyo^ presented in **a** *k*-space and **b** *R*-space, phase-shifted to the Fe–S scattering path. Experimental spectra are provided as solid black lines, and corresponding fits as red dashed lines. Spectra are *k*^3^-weighted, and the Fourier transform was performed for a *k*-range of 0–12 Å. Fits were performed using *k*^2,3^-weighting over ranges of *k* = 2–12 Å^−1^ and *R* = 1.5–4 Å

The Fe K-edge EXAFS of MoFe^pre^ and MoFe^lyo^ are provided in Fig. 5, and corresponding fitting parameters are provided in Table 3. In R-space, the spectral differences between MoFe^pre^ and MoFe^lyo^ appear more clearly. With the presently employed transformed k-range, the spectrum of MoFe^pre^ in *R*-space appears as a broad feature centered at ~ 2.3 Å, with an additional higher *R* feature around 3.7 Å. Based on previous EXAFS studies, the ~ 2.3 Å feature is dominated by a combination of Fe–S, Fe–Fe short, and to a lesser extent Fe–Mo scattering paths [42, 59]. Meanwhile, the feature at 3.71 Å arises from a long-range Fe–Fe scatterer. Moving to MoFe^lyo^, the formerly single amalgamation at ~ 2.3 Å appears as two distinct spectral features, namely a peak at ~ 2.2 Å with a shoulder at ~ 2.6 Å. In addition, the long-range Fe–Fe scattering peak is shifted to 3.65 Å. Despite these seemingly disparate differences in the *R*-space spectra, the fitting parameters of MoFe^pre^ and MoFe^lyo^ show only minor changes. Namely, *σ*^2^ of the Fe–Mo and short Fe–Fe scatterers increase, indicating a broader distribution of bond distances in the metallocofactors of MoFe^lyo^ relative to MoFe^pre^. Furthermore, the average Fe–S distance is mildly contracted (0.01 Å) while the first short Fe–Fe distance expands slightly (0.01 Å). However, these changes are still well within the margin of error of the fits.

**Table 3:** Fe K-edge EXAFS fitting parameters for MoFe^pre^ and MoFe^lyo^

	Path	*N*^a^	*σ*^2^ (10^–3^ Å^2^)	*R* (Å)	*E*_0_	*R*-factor
MoFe^pre^	Mo	0.2	5.0 (2.5)	2.69 (0.01)	7120 (1)	0.0026
	S	3.6	6.8 (0.3)	2.28 (0.01)		
	Fe short	2.53	7.2 (0.5)	2.62 (0.01)		
	Fe short	0.93		2.85 (0.02)		
	Fe long	0.8	3.2 (1.2)	3.71 (0.01)		
MoFe^lyo^	Mo	0.2	10.0 (0.2)	2.70 (0.01)	7120 (1)	0.0118
	S	3.6	6.7 (0.7)	2.27 (0.01)		
	Fe short	2.53	9.0 (1.5)	2.63 (0.02)		
	Fe short1	0.93		2.85 (0.05)		
	Fe long	0.8	5.4 (4.2)	3.71 (0.04)		

Statistical errors are provided for *σ*^2^ and *R* as determined from the fitting procedure. For both data sets, fits were performed using *k*^2,3^-weighting over ranges of *k* = 2–12 Å^−1^ and *R* = 1.5–4 Å

Between the Mo and Fe K-edge EXAFS, it appears that the metallocofactors of MoFe remain intact and generally do not exhibit any sign of considerable oxidation, reduction, or degradation. However, lyophilization does appear to increase heterogeneity in the iron–sulfur cluster environments, as evidenced by the increased disorder (*σ*^2^) of the fit Fe–Mo and Fe–Fe scattering paths.

To further investigate the viability of lyophilization as a storage and preparatory method for extracted MoFe and FeP, the acetylene reducing activity of the reconstituted proteins was tested. This was performed by comparing assays using (a) MoFe^pre^ with FeP^pre^, (b) MoFe^pre^ with FeP^rec^, (c) MoFe^rec^ with FeP^pre^, and (d) MoFe^rec^ with FeP^rec^. The resulting activities and relative quantities of produced C_2_H_4_ are provided in Table 4. A small reduction in activity is seen with FeP^rec^, which is reduced further to 92% when using MoFe^rec^. The lyophilized components together produce 85% of the original activity. Previous studies using a lyophilized preparation of MoFe reported recovery of 87% acetylene reducing activity following reconstitution, which is in reasonable agreement with the present results for both sets of assays involving MoFe^rec^. This reduction in the activity could be due to a small degree of protein aggregation upon reconstitution, and may be improved by further refinement of the lyophilization/reconstitution methodology.

**Table 4:** Acetylene reducing activity of MoFe and FeP prior to lyophilization, and following reconstitution of lyophilized protein into 20 mM Tris, 100 mM NaCl, 10 mM MgCl_2_, at pH 7.4

	mol C_2_H_2_/μmol MoFe	% C_2_H_4_
MoFe^pre^ + FeP^pre^	400 ± 20	100 ± 4
MoFe^pre^ + FeP^rec^	390 ± 60	97 ± 10
MoFe^rec^ + FeP^pre^	370 ± 30	92 ± 8
MoFe^rec^ + FeP^rec^	320 ± 10	85 ± 3

Assays were performed in triplicate over the course of 30 min

## Conclusions

Handling of protein samples for spectroscopic characterization is often challenging, potentially requiring buffer exchange and additional concentrating steps which invariably result in significant losses. In addition, spectroscopic methods requiring ultra-high vacuum conditions or extremely high concentrations impose strong limitations on possible sample preparations for protein systems. Lyophilization of proteins from volatile buffer systems offers a solution to these constraints, foregoing the need for tedious concentrating or solvent exchange steps and allowing concentrations far greater than the solubility limit to be achieved.

Here, we have utilized Mo and Fe K-edge EXAFS in parallel with X-band EPR to investigate the properties of the metallocofactors of isolated MoFe and FeP proteins in the lyophilized state. We have found that some distortions occur at the FeMoco cofactor which likely arise from disruption of hydrogen bonding and increased heterogeneity in the secondary environment of this cluster. This is evidenced by the increased rhombicity observed in the *S* = ^3^/_2_ EPR signal, and an apparent contraction of the average Mo–O/N distances, as determined from Mo EXAFS. Meanwhile, little change is observed in the Fe EXAFS and no additional signals indicative of oxidation appear in the EPR, supporting that both the FeMoco and P-cluster structures and oxidation states are retained through the lyophilization process. In addition, EPR measurements of FeP demonstrate that this process has little effect on either the oxidation state or electronic structure of the F-cluster. This is particularly surprising, given the exposed nature of this metallocofactor and its previously established sensitivity to varying solvent environments. Furthermore, the acetylene activity levels seen for reconstituted lyophilized MoFe and FeP demonstrate that the reactivity of both components can be restored from this state. This not only supports the viability of lyophilization for sample preparation and storage, but further supports our spectroscopic results that denaturation and distortion of the protein scaffolds themselves remains minimal. The combination of these results not only demonstrates lyophilization as a valuable means of investigating the resting state of Mo nitrogenase, but opens the gateway for further study of the mechanism of Mo nitrogenase through the generation of solid-state forms of catalytic intermediates, currently underway in our laboratories.

## Supplementary Information

Supplementary Information.

## Data Availability

Not applicable.
