# Supplementary material for: Preparation and spectroscopic characterization of lyophilized Mo nitrogenase
Source: J Biol Inorg Chem. 2020 Dec 30;26(1):81–91. doi: 10.1007/s00775-020-01838-4 (PMC8038959; doi:10.1007/s00775-020-01838-4)
Supplement: Supplementary file 1 — Supplementary Information. [file 775_2020_1838_MOESM1_ESM.pdf]

# Supplementary Information: Preparation and Spectroscopic Characterization of Lyophilized Mo Nitrogenase

Casey Van Stappen*, Laure Decamps, Serena DeBeer

Max-Planck Institute for Chemical Energy Conversion, Stiftstrasse 34-36, 45470 Mülheim an der Ruhr, Germany

email: casey.van-stappen@cec.mpg.de

**ORCID**
Casey Van Stappen: 0000-0002-1770-2231
Laure Decamps: 0000-0002-4572-7479
Serena DeBeer: 0000-0002-5196-3400

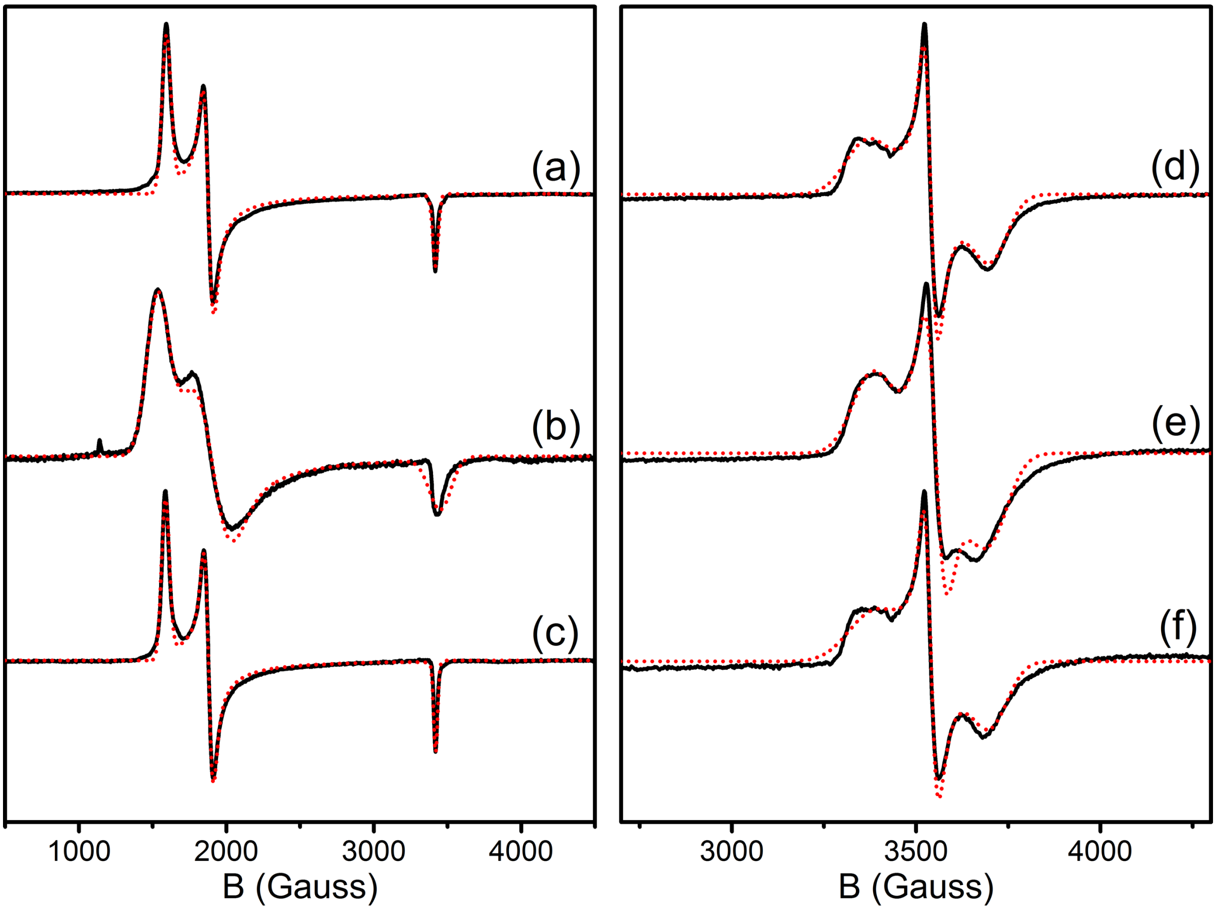


**Fig. 1** X-band CW perpendicular-mode EPR spectra fits of (left) MoFe and (right) FeP. a) $MoFe^{pre}$, b) $MoFe^{lyo}$, c) $MoFe^{rec}$, d) $FeP^{pre}$, e) $FeP^{lyo}$, and f) $FeP^{rec}$. Experimental data are provided as solid black lines, and fits as red dashed lines. Measurements were performed at 10 K, 9.6 GHz using a power of 2 mW and a 7.46 G modulation amplitude.
